# 549. A Phase 2a Randomized, Double-Blind, Controlled Trial of the Efficacy and Safety of an Intravenous (IV) Bacteriophage Cocktail (AP-SA02) vs. Placebo in Combination with Best Available Antibiotic Therapy (BAT) in Patients with Complicated Staphylococcus aureus Bacteremia

**DOI:** 10.1093/ofid/ofaf695.022

**Published:** 2026-01-11

**Authors:** Loren G Miller, Stacey Kolar, John Sanders, John Williamson, Paul R Allyn, Jihoon Baang, Paul F Riska, Saima Aslam, George J Alangaden, Jane Wainaina, Colleen S Kraft, Nirja Mehta, Deena Altman, Gary P Wang, Mehdi Mirsaeidi, D Alexander Perry, Jose A Vazquez, Lindsay Nicholson, Jonathan Iredell, Pierre Kyme, Deborah Birx, Vance G Fowler

**Affiliations:** David Geffen School of Medicine at UCLA, Torrance, California; Armata Pharmaceuticals, Los Angeles, California; Wake Forest University, Winston, North Carolina; Wake Forest University, Winston, North Carolina; UCLA, Los Angeles, California; University of Michigan Medical Center, Ann Arbor, Michigan; Montefiore Medical Center, Bronx, New York; Univesity of California at San Diego, San Diego, California; Henry Ford Health, Detroit, Michigan; Froedtert Hospital & Medical College of Wisconsin, Milwaukee, Wisconsin; Emory University, Atlanta, GA; Emory University, Atlanta, GA; Icahn School of Medicine at Mount Sinai, New York, New York; University of Florida, Gainesville, Florida; University of Florida Health - Jacksonville, Jacksonville, Florida; University of Arizona - Banner Health, Tucson, Arizona; Medical College of Georgia at Augusta University, Augusta, GA; Rocky Mountain Regional Veterans Administration Medical Center, Aurora, Colorado; Microbial Genomics Reference Laboratory, Centre for Infectious Diseases and Microbiology Laboratory Services, NSW Health Pathology, ICPMR Westmead, NSWAustralia; University of Sydney, Sydney, NSW Australia; Centre for Infectious Diseases and Microbiology, The Westmead Institute for Medical Research, Westmead Hospital, NSW Australia, Westmead, New South Wales, Australia; Armata Pharmaceuticals, Los Angeles, California; Armata Pharmaceuticals, Los Angeles, California; Duke University Medical Center, Durham, NC

## Abstract

**Background:**

Complicated *Staphylococcus aureus* bacteremia (SAB) is a serious, common, and frequently lethal infection. Treatment options are complicated by resistance, drug intolerance, or relapse. Novel therapeutics are urgently needed.

**Figure:**
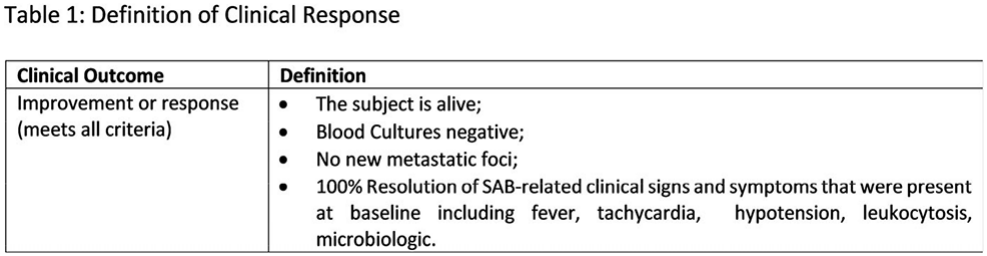

**Figure:**
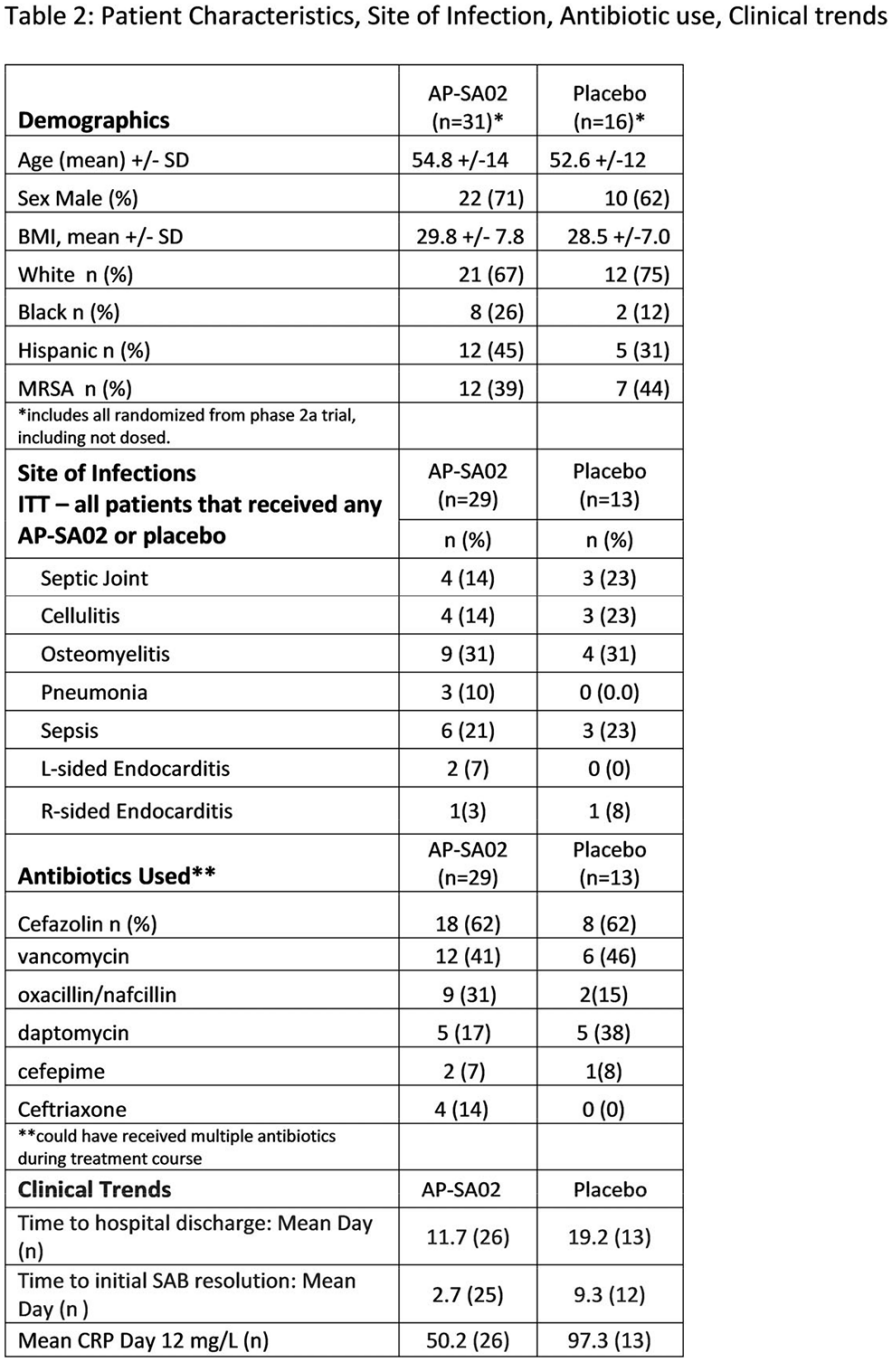

**Methods:**

We performed a phase 2a randomized, double-blind, controlled trial of the efficacy and safety of an IV bacteriophage cocktail, AP-SA02, q6 hrs x 5 days vs. placebo (2:1 ratio) in combination with BAT in patients with complicated SAB. Clinical response (Table 1) was assessed in the intent-to-treat (ITT) population at Test of Cure (TOC) on Day 12, post-BAT, and End of Study (EOS) four weeks after BAT completion. Safety analysis included data from the Phase 1b trial (n=8).

**Figure:**
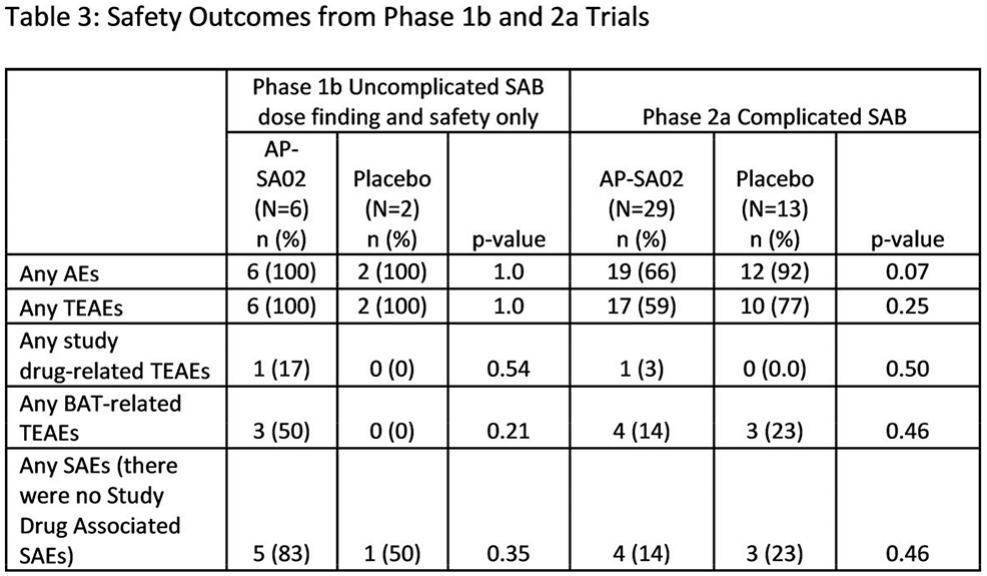

**Figure:**
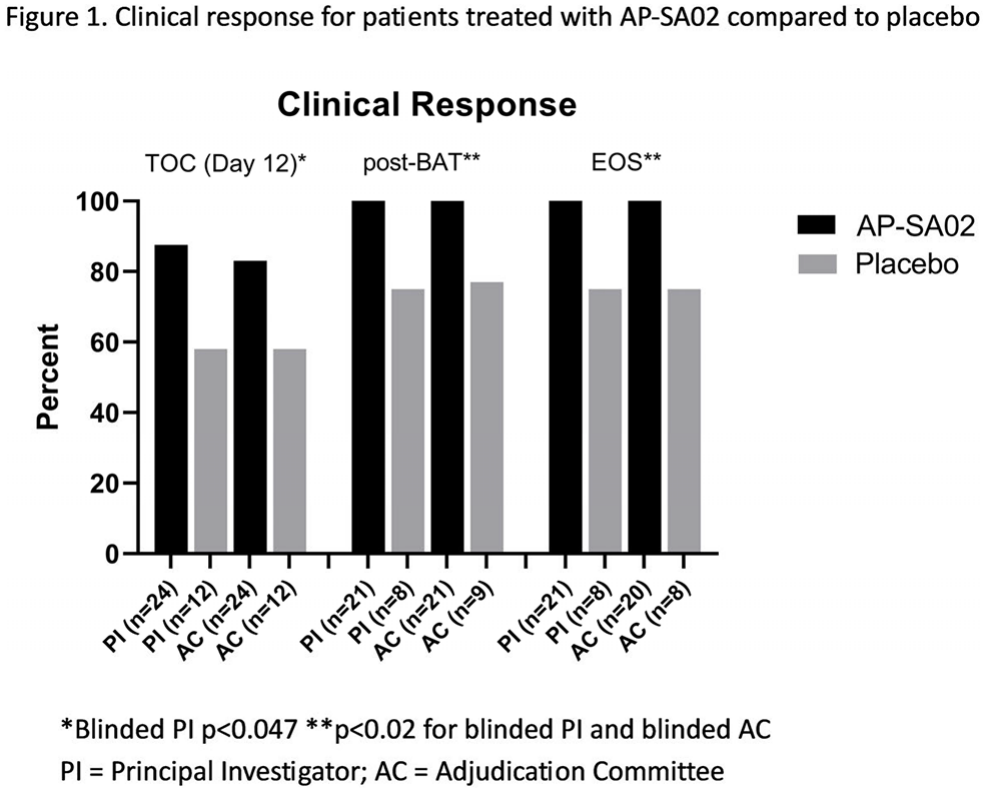

**Results:**

We enrolled 42 patients from 17 sites (95% US), with 29 randomized to AP-SA02 (A) and 13 to placebo (P). MRSA was the pathogen in 39% of the (A) and 44% (P) groups, respectively. Site of infection and antibiotics used were similar for both arms. (Table 2). Treatment-emergent adverse events (TEAEs) occurred in 6% (2/35) and 0% (0/15) in the (A) and (P) groups, respectively. (Table 3). Day 12 clinical response rates were 88% (21/24) in SA-PA02 vs. 58% (7/12) in the placebo (p = 0.047) as assessed by blinded site investigators (PI), and 83% (20/24) vs. 58% (7/12) as assessed by the blinded Adjudication Committee (AC). At post-BAT and EOS, non-response/relapse rate was 0% and 0% in the (A) group by both PI and AC assessment compared to 23% by AC and 25% PI in the (P) group (p < 0.025, Figure 1). Patients on AP-SA02 had trends toward rapid normalization of C-reactive protein, shorter time to negative blood culture, shorter ICU and hospital stay. (Table 2)

**Conclusion:**

The intravenous bacteriophage cocktail, AP-SA02, combined with BAT, had a higher and earlier cure rate compared to placebo in patients with complicated SAB at day 12, at post BAT, and EOS as assessed by both blinded site investigators and independent adjudicators. AP-SA02 appears safe with clinical efficacy against both MRSA and MSSA and trends toward earlier resolution, shorter hospitalization, and no evidence of relapse 4 weeks post-therapy. Results strongly support proceeding to a Phase III trial of this novel bacteriophage cocktail for SAB.

**Disclosures:**

All Authors: No reported disclosures

